# The relationship between food quality score with inflammatory biomarkers, and antioxidant capacity in young women

**DOI:** 10.14814/phy2.15590

**Published:** 2023-01-25

**Authors:** Afsane Bahrami, Fatemeh Nikoomanesh, Zahra Khorasanchi, Malihe Mohamadian, Gordon A. Ferns

**Affiliations:** ^1^ Clinical Research Development Unit, Imam Reza Hospital, Faculty of Medicine Mashhad University of Medical Sciences Mashhad Iran; ^2^ Clinical Research Development Unit of Akbar Hospital Mashhad University of Medical Sciences Mashhad Iran; ^3^ Infectious Diseases Research Center Birjand University of Medical Sciences Birjand Iran; ^4^ Cellular and Molecular Research Center Birjand University of Medical Sciences Birjand Iran; ^5^ Department of Nutrition, School of Medicine Mashhad University of Medical Sciences Mashhad Iran; ^6^ Division of Medical Education Brighton & Sussex Medical School Brighton UK

**Keywords:** inflammation, MDA, neutrophil, oxidative stress

## Abstract

Diet has the potential to decrease oxidative stress and inflammation and this may be beneficial in several diseases. This study investigated the association between food quality score (FQS) with antioxidant and inflammatory properties in 171 apparently healthy young women. This cross‐sectional study was conducted using a validated food frequency questionnaire to determine the dietary intake of participants. FQS was calculated by summing all the scores obtained from healthy and unhealthy food groups. The total antioxidant capacity and free radical scavenging activity of serum and urine were quantified using the ferric reducing/antioxidant power (FRAP) and α, α‐diphenyl‐β‐picrylhydrazyl (DPPH) methods, respectively. Malondialdehyde (MDA) was measured using the formation of thiobarbituric acid reactive substances (TBARS). White blood cell (WBC) and neutrophil counts, mean platelet volume (MPV) and red blood cell distribution width (RDW), were measured. Neutrophil: lymphocyte ratio (NLR), platelet: lymphocyte ratio (PLR), and RDW: platelet ratio (RPR) were also calculated. A high food quality (rich in fruit and vegetables, nuts, whole grain, and low intake of sweetened beverage, potato chips and fried food from outside the home) was related to lower hematological inflammatory biomarkers including WBC count, RDW, NLR, and PLR. Multivariable‐adjusted odds ratios (95% CIs) demonstrated that higher FQS group (third tertile vs. first tertile) was associated with a significant lower levels of urinary FRAP (OR_adj_ = 0.82; 95%CI: 0.70 to 0.97), and DPPH. High food quality was associated with reduced of markers of inflammation and oxidative stress in Iranian young girl.

## Graphical abstract

Diet has the potential to decrease oxidative stress and inflammation and this may be beneficial in different diseases. Our findings demonstrated that high food quality was associated with reduced markers of inflammation and oxidative stress in Iranian young girl.

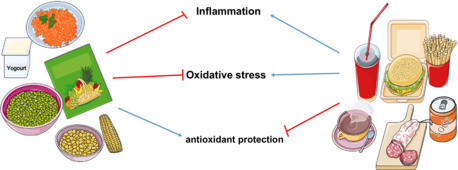

## 1 INTRODUCTION

It has been well established that diet quality is closely related to mortality and morbidity particularly from chronic diseases (Lee et al., 2011). An adequate intake of macro and micronutrients is required for rapid physical growth, maturity, and cognitive development in childhood and adolescence (Ushula et al., 2021). First and foremost, to improve dietary patterns (DPs), it is necessary to investigate the factors that influence dietary behaviors and how these factors influence diet quality. Dietary guidelines place a strong emphasis on the promotion of good nutrition and health (Thorpe et al., 2014).

The assessment of single nutrient intakes does not accurately reflect the overall diet quality, and hence some dietary scores, like the Food‐Based Diet Quality Score (FQS), have been developed. Nutrient intake assessments do not require a database or software, and food‐based scores are easily adjusted for clinical use (Waijers et al., 2007). FQS is usually determined by summing up different food groups that fall into two categories: healthy and unhealthy (Darooghegi Mofrad et al., 2019). FQS has previously been assessed in a limited number of studies where its association with the risk of metabolic syndrome (Lavigne‐Robichaud et al., 2018), coronary heart disease (Fung et al., 2016), cardiovascular disease (CVD; Darooghegi Mofrad et al., 2019), and breast cancer (Hosseini et al., 2021) has been evaluated.

Oxidative stress and inflammation are believed to be involved in several chronic diseases. Oxidative stress is defined as the homeostatic imbalance between oxygen‐derived metabolites, predominantly reactive oxygen species (ROS), and the potential of cellular antioxidant protection systems. Higher levels of ROS formation is closely related to oxidative stress. Oxidative damage of biomolecules is contributed to the etiology, development, and pathogenesis of a wide spectrum of chronic disorders, such as neurodegenerative, cardiovascular, autoimmune, tumors, lung, digestive, and inflammatory, among others, and plays a considerable role in the aging of cells (Kruk et al., 2019; Bandyopadhyay et al., 1999).

There is growing evidence that systemic inflammatory responses can be evaluated by hematological parameters that are easily measured in the clinic; for example white blood cells (WBC), neutrophils, red blood cell distribution width (RDW), mean platelet volume (MPV), neutrophil/lymphocyte ratio (NLR), platelet/lymphocyte ratio (PLR), and RDW/platelet ratio (RPR; Fu et al., 2015; Hu et al., 2013; Yang et al., 2017). The predictive or prognostic value of these parameters has been studied in a variety of conditions such as cancers (Wang et al., 2017; Bao et al., 2018), CVDs (Kamińska et al., 2018), autoimmune (Yang et al., 2015), and parasitic diseases (Hu et al., 2019).

Diet quality has the potential to decrease oxidative stress and inflammation that may be beneficial (Kim et al., 2011; Bärebring et al., 2018). For instance, the consumption of various fruits and vegetables elevated the antioxidant capacity (TAC) of serum, saliva, and urine (Hassimotto et al., 2008; Cao et al., 1998; Jacob et al., 2008; Stewart et al., 2002). However, another study reported that there was no significant effect of following a specific dietary pattern on oxidative stress markers in healthy individuals (Miller III et al., 2005). Bärebring et al. (2018) have reported that high dietary quality is inversely associated with inflammatory markers such as high sensitivity C‐reactive protein (hs‐CRP) and erythrocyte sedimentation rate (ESR). A recent systematic review analysis found that plant‐based DPs are linked to reduced levels of inflammation and oxidative stress biomarkers, suggesting preventive approaches to chronic disease (Aleksandrova et al., 2021).

To our knowledge, no comprehensive evidence currently found to inspect the relationship between FQS and inflammatory status, and antioxidant capacity. With respect to the great importance of DPs in well‐being and health, we performed a cross‐sectional survey to investigate the association between FQS with antioxidant and inflammatory properties of the serum and urine of apparently healthy young women.

## 2 MATERIALS AND METHODS

### 2.1 Study design

The study was undertaken in Birjand, in northwestern of Iran, in January 2020 among female university student aged between 18 and 24 years old (Abbaszadeh et al., 2020, Askari et al., 2020). A total of 330 females were initially screened for inclusion. We excluded those having any acute or chronic complications or who were taking medication or nutritional supplements. The final population sample comprised 171 healthy young women who were recruited from five different universities in Birjand using a multistage cluster sampling method.

Since we aimed to perform our investigation on a homogeneous population, in order to control for potential confounders, only single, apparently healthy women were included. The Ethical Committee of our university approved the study, and informed written consent was obtained from all participants.

### 2.2 Adherence to FQS

A validated food frequency questionnaire (FFQ) was employed to estimate the food intake of individuals (Ahmadnezhad et al., 2017, Abbaszadeh et al., 2020). A proficient dietitian instructed study volunteers to report their food intake frequency for each item within the last year on per day, week, month, rarely or never basis. Food analysis was performed using Diet Plan 6 software (forest field Software Ltd). Food quality scoring was performed by the scale developed by Fung et al. (2016). FQS ingredients include vegetables, fruits, whole grains, nuts and legumes, yogurt, coffee as healthy foods as well as refined grains, sugar‐sweetened beverages, desserts and ice cream, red and processed meats, potato and potato chips, and fried food consumed outside the home as unhealthy foods. We then classified the participants' intake into decile. The value between 1 and 10 was assigned to each healthy component. For unhealthy components, a reverse scoring process (values between 10 and 1) was assigned. Finally, the total FQS (in the range of 14–140) was calculated by summing all the scores obtained for each participant, so that a higher score indicates a healthier diet.

### 2.3 Sampling

The blood and urine samples and FFQ data were collected on the same day. Blood and urine samples were collected after a 12 h fasting. The volunteers were instructed to avoid intense physical activity 24‐h before the sampling. Blood samples were collected into both serum separator tubes and EDTA tubes. Sterile disposable container was used for collecting urine specimens from the first‐morning section of urine from the middle stream according to a standard protocol. Serum and urine specimens were stored at −80°C until analysis.

### 2.4 Biochemical measurements

The serum concentrations of, urea, creatinine, alanine transaminase (ALT), aspartate transaminase (AST), alkaline phosphatase (ALP), total bilirubin, direct bilirubin, total protein, albumin, calcium, phosphate, magnesium and uric acid, fasting blood glucose (FBG) and hs‐CRP were quantified using commercial kits (Pars Azmun) and an auto‐analyzer (Prestige 24i, Tokyo Boeki Ltd.).

### 2.5 Complete blood count

Blood cell counts, hemoglobin levels, dimensional variables (MPV and RDW), and some combined parameters, such as NLR, PLR and RPR, were assessed by means of an automated commercial cell counter (Sysmex K‐800).

### 2.6 Serum and urine oxidative stress parameters

#### 2.6.1 Total antioxidant capacity (TAC)

TAC was quantified using the ferric reducing/antioxidant power (FRAP) method (Benzie and Strain, 1996). This procedure is based on the reduction of a ferric–tripyridyl triazine (Fe^3+^‐TPTZ) complex to its ferrous (Fe^2+^) colored appearance in the presence of antioxidant compounds. The tests were carried out in 260 μl reaction mixtures including 250 μl of FRAP solution plus 10 μl serum, standard (FeSO_4_) and blank (for each sample, a blank sample was used to remove turbidity). The absorbance was measured colorimetrically at 593 nm. All tests were undertaken in duplicate and TAC measure of samples was described in μmol TAC/L. For urine samples, the samples diluted 1:10 and the findings are demonstrated in μmol TAC/mg creatinine.

#### 2.6.2 Free radical scavenging activity

The free radical scavenging activity in samples was assessed using the A‐Diphenyl‐B‐Picrylhydrazyl (DPPH) method (Janaszewska and Bartosz, 2002). Tests were conducted in reaction mixtures including 1 ml of 100 mM DPPH solution and 40 μl of each serum and blank samples. After incubation at room temperature for 10 min, each sample was centrifuged at 4000 *g* for 5 min at 37°C to remove cells. Absorbance was read using a microplate reader (Epoch‐Biotek®) at 517 nm and compared with that of blank samples, including only DPPH and methanol solution. The results are reported in mmol Trolox equivalent/L. The urine tests were performed in reaction mixes including 250 μl of DPPH reagent and 10 μl urine (the samples diluted 1:10) and blank samples. The results are shown in mmol Trolox equivalent/mg creatinine.

#### 2.6.3 Malondialdehyde (MDA) assay

The thiobarbituric acid‐reactive‐substances (TBARS), an index of lipid peroxidation, was measured using the method described by Kei (1978). The end product of fatty acid peroxidation, malonyldialdehyde (MDA), interacts with TBA to generate a colored complex. TBARS reagent (1 ml) was mixed with samples (100 μl), and the admixture was heated in a boiling water bath for 20 min. Next, 1 ml of N‐butanol was used to extract TBARS adducts and solution centrifuged at 1500 *g* (10 min at 4°C). The supernatant was collected and record the fluorescence intensity at excitation and, emission wavelengths of 515 and 553 nm. Findings were quantified by comparing them to the standard curve obtained from standard solutions under the same conditions. The TBARS concentration of samples is expressed in μmol TBARs/L. For urine samples the results are presented in μmol TBARs/mg creatinine.

### 2.7 Other variables

Anthropometric parameters as well as blood pressure were measured according to standard instruction (Ho et al., 2001).

### 2.8 Statistical analysis

Kolmogorov–Smirnov tests were recruited to evaluate the normality of the distribution of variables. Subjects were sub‐grouped into three categories according to tertiles of their FQS. One‐way analysis of the variance (ANOVA) test (normal distribution parameters) or Kruskal–Wallis test (non‐normal distribution parameters) were employed for comparison of quantitative variables between tertiles of FQS. Moreover we performed a multivariable logistic regression to explore the relationship between FQS with inflammatory and oxidative stress parameters using the FQS tertile groups as dependent variable (reference category: first tertile), after correction for energy intake, WHR, total protein and phosphate levels. All statistical analyses were undertaken using the SPSS version 16.0 and *p* values ≤0.05 were set as significant.

## 3 RESULTS

The FQS were used for categorizing of the participants into tertiles, with T1 set as the bottom tertile (lower score; range: 19–26; *n* = 55), T2 (middle score; range: 27–29; *n* = 57), and T3 as top tertile (higher score; range: 30–35; *n* = 59). Kolmogorov–Smirnov test indicated a normal distribution of all variables (*p* > 0.05), except serum hs‐CRP and triglycerides.

No significant difference was found between the participants across tertiles of FQS regarding to demographic and anthropometric parameters, lipid profile, liver function enzyme tests, FBG, and albumin (*p* > 0.05; Table 1). However, WHR were lower in the third tertile than in the second and first tertile (*p* = 0.007). Individuals in the highest tertile of the FQS tended to have lower serum levels of total protein and phosphate compared with those in the lowest tertiles (*p* < 0.05; Table 1).

**TABLE 1:** Baseline clinical characteristics of study participants (*n* = 171)

	Tertiles of FQS			*p* value
	T1 (*n* = 55)	T2 (*n* = 57)	T3 (*n* = 59)	
FQS, score	24.4 ± 1.6	28.1 ± 0.8	31.3 ± 1.3	**<0.**001^a^ ^,^ ^b^ ^,^ ^c^
Age, year	21.3 ± 2.0	20.7 ± 1.8	20.6 ± 1.5	0.11
BMI (kg/m^2^)	20.5 ± 2.3	21.7 ± 2.9	20.9 ± 3.1	0.18
WHR	0.75 ± 0.04	0.73 ± 0.04	0.72 ± 0.03	**0.007** ^a^
SBP (mmHg)	106 ± 84	106 ± 87	108 ± 103	0.59
DBP (mmHg)	71 ± 63	70 ± 74	71 ± 86	0.83
HDL‐C (mg/dl)	50.9 ± 7.8	49.6 ± 10.0	50.9 ± 8.9	0.75
LDL‐C (mg/dl)	72.6 ± 18.9	67.7 ± 15.9	74.3 ± 16.7	0.47
TG (mg/dl)	66.0 (48.0–85.2)	69.0 (59.0–102.0)	62.0 (52.0–85.0)	0.12
TC (mg/dl)	154 ± 28	149 ± 22	152 ± 24	0.57
FBG (mg/dl)	84.3 ± 6.8	82.7 ± 6.3	84.0 ± 7.3	0.55
Urea(mg/dl)	29.4 ± 6.0	29.0 ± 7.3	31.1 ± 8.0	0.35
Creatinine(mg/dl)	1.0 ± 0.5	0.97 ± 0.13	1.0 ± 0.44	0.73
ALT (IU/L)	20.6 ± 12.2	20.2 ± 11.2	19.7 ± 14.4	0.94
AST (IU/L)	16.2 ± 8.8	17.6 ± 15.7	15.8 ± 11.9	0.79
ALP (IU/L)	187.8 ± 39.9	177.9 ± 34.7	197.7 ± 43.3	0.07
Direct bilirubin (mg/dl)	0.31 ± 0.13	0.30 ± 0.16	0.29 ± 0.14	0.94
Total bilirubin (mg/dl)	0.63 ± 0.27	0.61 ± 0.31	0.63 ± 0.30	0.98
Total protein (g/day)	8.1 ± 0.49	7.9 ± 0.44	7.9 ± 0.38	**0.028** ^a^ ^,^ ^c^
Albumin (g/dl)	5.1 ± 0.29	5.0 ± 0.26	5.0 ± 0.29	0.36
Calcium(mg/dl)	10.2 ± 0.49	10.1 ± 0.43	10.0 ± 0.46	0.32
Phosphate(mg/dl)	5.4 ± 0.70	5.1 ± 0.59	5.0 ± 0.65	**0.028** ^a^
Magnesium(mg/dl)	2.4 ± 0.32	2.3 ± 0.21	2.3 ± 0.26	0.22
Uric acid (mg/dl)	3.1 ± 0.88	3.3 ± 0.83	3.0 ± 0.57	0.10

*Note*: Data presented as Mean ± SD or median (interquartile range). By using ANOVA and post hoc Tukey or Kruskal–Wallis. Significance of bold values were *p* < 0.05.

Abbreviations: ALP, alkaline phosphatase; ALT, alanine transaminase; AST, aspartate transaminase; BMI, body mass index; DBP, diastolic blood pressure; HDL‐C, high‐density lipoprotein cholesterol; LDL‐C, low‐density lipoprotein cholesterol; SBP, systolic blood pressure; TC, total cholesterol; TG, triglyceride; WHR, waist to hip ratio.

^a^ Significant between tertile 1 and 3.

^b^ Significant between tertile 2 and 3.

^c^ Significant between tertile 1 and 3.

The dietary intake of subjects across tertiles of FQS assessments was shown in Table 2. The subjects with a higher tertile of FQS had a greater consuming of dietary fiber, MUFA, vitamin B6, vitamin C, magnesium, Fe, vegetables, fruits, legumes and nuts, and whole grains compared to lowest tertile. Furthermore, eating of fat, sugar sweetened beverage, potato chips, and fried food from outside the home higher more in participants who were placed in the third tertile of FQS versus to the first tertile (*p* < 0.05; Table 2).

**TABLE 2:** Comparison of dietary intakes of participants between tertiles of the adherence to the FQS

Variables	FQS tertiles			*p* value
	T1 (*n* = 55)	T2 (*n* = 57)	T3 (*n* = 59)	
Nutrient (per day)				
Energy (kcal)	2190 ± 733	2320 ± 795	2027 ± 784	0.14
Protein (g)	32.4 ± 17.0	30.3 ± 12.1	33.8 ± 17.0	0.51
Carbohydrate (g)	65.4 ± 27.5	58.7 ± 31.8	61.5 ± 35.8	0.58
Fat (g)	19.2 ± 10.4	13.9 ± 11.2	12.2 ± 12.5	**0.004**
Dietary fiber (g)	11.6 ± 4.2	12.0 ± 4.9	15.8 ± 7.4	**<0.001**
SFA (g)	17.1 ± 8.6	17.5 ± 8.1	19.3 ± 10.9	0.43
MUFA(g)	15.4 ± 10.7	14.1 ± 9.4	24.2 ± 26.2	**0.007**
PUFA (g)	29.8 ± 21.5	35.9 ± 28.4	32.9 ± 25.0	0.58
Vitamin B6 (mg)	0.91 ± 0.38	1.0 ± 0.27	1.3 ± 0.47	**<0.001**
Vitamin B12 (μg)	2.9 ± 1.8	2.7 ± 1.3	2.4 ± 1.7	0.28
Vitamin C (mg)	75.4 ± 60.1	97.1 ± 68.6	181.6 ± 146.6	**<0.001**
Calcium (mg)	437 ± 295	477 ± 245	369 ± 182	0.062
Magnesium (mg)	155.0 ± 60.9	192.2 ± 95.9	219.9 ± 75.3	**<0.001**
Zinc (mg/)	4.7 ± 2.3	5.0 ± 1.8	4.2 ± 1.6	0.08
Fe (mg)	5.7 ± 2.0	6.1 ± 2.8	7.0 ± 2.3	**0.013**
Food groups (g/day)				
Vegetables	70.2 ± 60.7	104.1 ± 90.1	172.9 ± 169.1	**<0.001**
Fruits	84.8 ± 104.9	118.2 ± 126.6	251.2 ± 259.8	**<0.001**
Legumes and nuts	19.4 ± 22.7	45.2 ± 37.0	54.9 ± 42.5	**<0.001**
Whole grains	21.9 ± 30.3	26.6 ± 49.1	41.8 ± 47.8	**0.04**
Yogurt	38.9 ± 90.7	51.0 ± 54.1	40.6 ± 53.5	0.64
Sugar sweetened beverage	103.1 ± 123.3	60.0 ± 123.2	32.8 ± 75.9	**0.007**
Red meat	41.3 ± 38.2	38.6 ± 28.8	35.4 ± 47.4	0.73
Refined grains	206.3 ± 155.8	170.0 ± 190.2	162.2 ± 110.3	0.30
Desserts and ice cream	15.8 ± 24.0	15.0 ± 20.3	16.8 ± 29.9	0.93
Potato	35.3 ± 44.5	40.7 ± 51.0	40.8 ± 47.3	0.80
Potato chips	13.9 ± 29.9	9.9 ± 10.1	3.3 ± 4.9	**0.018**
Coffee	15.7 ± 45.8	15.9 ± 32.7	8.6 ± 34.4	0.51
Fried food from outside the home	101.3 ± 124.2	45.9 ± 65.9	45.0 ± 86.6	**0.004**

*Note*: Data presented as Mean ± SD and adjusted for energy intake. T1 represents low compliance and T3 a high compliance with a FQS. Significance of bold values were *p* < 0.05. *p*‐Value obtained from ANOVA.

Abbreviations: MUFA, monounsaturated fatty acid; PUFA, polyunsaturated fatty acid; SFA, saturated fatty acid.

Additionally, individuals in higher group of FQS, were more likely to have lower blood WBC, RDW, NLR, PLR as well as urinary FRAP and DPPH levels (*p* < 0.05). Serum FRAP was found to be lower in the bottom than in the top tertile of the FQS (*p* = 0.030; Table 3).

**TABLE 3:** Inflammatory profile and anti‐oxidant status of individuals across FQS tertiles categories

	Tertiles of FQS			*p*
	T1 (*n* = 55)	T2 (*n* = 57)	T3 (*n* = 59)	
Inflammatory parameters				
WBC (10^9^ cells/L)	7.3 ± 2.4	7.1 ± 1.5	6.4 ± 1.4	**0.026** ^a^
Neutrophil (%)	54.4 ± 11.8	53.3 ± 7.2	52.0 ± 10.3	0.51
RDW (%)	13.6 ± 1.5	13.1 ± 0.85	13.0 ± 0.81	**0.040** ^a^
MPV(fL)	10.3 ± 1.0	10.3 ± 0.83	10.3 ± 0.93	0.98
NLR	1.8 ± 1.3	1.5 ± 0.56	1.2 ± 0.42	**0.048** ^a^
PLR	8.5 ± 4.1	7.6 ± 2.4	6.6 ± 1.9	**0.011** ^a^ ^,^ ^b^
RPR	0.05 ± 0.01	0.04 ± 0.01	0.05 ± 0.01	0.36
hsCRP (mg/L)	0.2 (0.01–0.37)	0.4 (0.01–1.5)	0.4 (0.1–1.0)	0.051
Anti‐oxidant status				
Urinary FRAP (μmol TAC/ mg Cr)	6.0 ± 2.2	8.7 ± 7.6	11.1 ± 10.2	**0.041** ^a^ ^,^ ^b^
Urinary DPPH (mmol trolox equivalent/mg Cr)	1.5 ± 0.71	2.3 ± 2.2	2.5 ± 1.5	**0.032** ^a^
Urinary MDA (μmol TBARs/mg Cr)	0.87 ± 0.75	0.81 ± 0.79	0.83 ± 0.65	0.96
Serum FRAP (μmol TAC/L)	647 ± 82	671 ± 126	716 ± 144	**0.030** ^a^
Serum DPPH (mmol trolox equivalent/L)	75.8 ± 59.1	94.3 ± 62.7	86.2 ± 54.4	0.38
Serum MDA (μmol TBARs/L)	0.57 ± 0.22	0.77 ± 0.57	0.70 ± 0.28	0.30

*Note*: Data presented as Mean ± SD or median (IQR). Significance of bold values were *p* < 0.05. Obtained from ANOVA test and post hoc Tukey.

Abbreviations: Cr, creatinine; DPPH, α, α‐diphenyl‐β‐picrylhydrazyl; FRAP, Ferric reducing/antioxidant power; hsCRP, high‐sensitivity C‐reactive protein; MDA, malondialdehyde; MPV, mean platelet volume; NLR, neutrophil: lymphocyte ratio; PLR, platelet: lymphocyte ratio; RDW, red blood cell distribution width; RPR, red blood cell distribution width: platelet ratio; WBC, White blood cell.

^a^ Significant between tertile 1 and 3.

^b^ Significant between tertile 2 and 3.

Multivariable‐adjusted ORs (95% CIs) after controlling of energy intake, WHR, total protein and phosphate levels that higher FQS group (third tertile vs. first tertile) was associated with a significant lower levels of blood WBC (OR_adj_ = 0.71; 95%CI: 0.54 to 0.92), RDW (OR_adj_ = 0.63; 95%CI: 0.39–0.99), NLR (OR_adj_ = 0.51; 95%CI: 0.27 to 0.99), PLR (OR_adj_ = 0.77; 95%CI: 0.65 to 0.93), urinary FRAP (OR_adj_ = 0.82; 95%CI: 0.70 to 0.97), and urinary DPPH (OR_adj_ = 0.59; 95%CI: 0.36 to 0.97; Table 4).

**TABLE 4:** Adjusted OR and 95% confidence interval for inflammatory, antioxidant and oxidative parameters between tertiles of FQS scores

Parameters	Crude			Adjusted		
	T1	T2	T3	T1	T2	T3
WBC	Ref.	0.94 (0.73–1.18)	0.69 (0.52–0.91)**	Ref.	0.91 (0.71‐1.5)	0.71 (0.54–0.92)**
RDW	Ref.	0.67 (0.42–1.01)	0.63 (0.39–0.99)*	Ref.	0.68 (0.46‐1.02)	0.64 (0.39–0.99)*
NLR	Ref.	0.70 (0.40–1.21)	0.49 (0.25–0.95)*	Ref.	0.71 (0.42‐1.25)	0.51 (0.27–0.99)*
PLR	Ref.	0.92 (0.79–1.07)	0.78 (0.65–0.92)**	Ref.	0.90 (0.78‐1.05)	0.77 (0.65–0.93)**
Urinary FRAP	Ref.	1.02 (0.96–1.08)	0.83 (0.71–0.96)*	Ref.	1.01 (0.96‐1.07)	0.82 (0.70–0.97)*
Urinary DPPH	Ref.	1.03 (0.92–1.3)	0.59 (0.36–0.98)*	Ref.	1.04 (0.83‐1.3)	0.59 (0.36–0.97)*
Serum FRAP	Ref.	1.00 (0.99–1.007)	0.99 (0.99–1.00)	Ref.	1.00 (0.99–1.006)	0.99 (0.99–1.00)

*Note*: Tertile 1 was considered as reference group. Adjusted for energy intake, WHR, total protein and phosphate levels.

Abbreviations: DPPH, α, α‐diphenyl‐β‐picrylhydrazyl; FRAP, ferric reducing/antioxidant power; NLR, neutrophil: lymphocyte ratio; PLR, platelet: lymphocyte ratio; RDW, red blood cell distribution width; WBC, White blood cell.

* *p* < 0.05

** *p* < 0.01.

## 4 DISCUSSION

Findings from this cross‐sectional survey provide evidence that a high FQS (with a high intake of fruit and vegetables, nuts, whole grain, and low intake of sweetened beverage, potato chips and fried food from outside the home) was related to lower levels of inflammatory biomarkers including WBC count, RDW, NLR, and PLR. Higher FQS also associated to reduction of urinary FRAP, DPPH levels. To our knowledge, this is the first attempt investigating the association between FQS and biomarkers of inflammation and anti‐oxidant capacity in healthy young women.

We also found that a high FQS was associated with a high intake of antioxidant‐rich foods, which might reduce oxidative stress. We found that young healthy women with a high FQS have significantly lower urinary concentrations of FRAP, and DPPH. In Korean population, two food quality scales, Recommended Food Score (RFS) and the alternate Mediterranean Diet Index (aMED), were negatively associated with urinary MDA concentrations (Kim et al., 2011). The eating of vegetables, particularly green leaves, and fruits connected with lower oxidative stress (Paz et al., 2019). Interestingly, a Western dietary style identified by the intake of components with a high glycemic index may causes to impaired blood glucose homeostasis, and elevate oxidative stress (Liu et al., 2002). There is inconsistent evidence on the association between dietary fruit and vegetables and urinary markers of oxidative stress. Hassimotto et al. (2008) found reduced urinary TAC concentrations after a single ingestion of blackberry juices. Tsang et al. (2012) announced the elevation in plasma, but not urinary FRAP levels following fruit juice intake. Urinary TAC was also unaltered post 3 weeks consuming of a antioxidants fortified dried fruit and vegetable (Stewart et al., 2002). Vitamin C and magnesium reduce NADPH oxidase as a superoxide‐producing enzyme, which supports that increasing antioxidant capacity can lead to the attenuation of ROS (Lopes et al., 2003, Chen et al., 2001). It has been reported that FRAP levels were proportional to the mitigating power of the major non‐enzymatic antioxidants in serum (Benzie and Strain, 1996); so, this assay was chosen to evaluate the antioxidant status of young women in the present study. We found serum FRAP was lower in the bottom than in the top tertile of the FQS; this finding indicates that the better diet quality causes to increased non‐enzymatic antioxidant defenses.

Oxidative stress is related with an imbalance between free radical generation and antioxidant defenses throughout the body, it is usually necessary to assess the counterpart of oxidation, the total antioxidant status. Additional indices suggest an association between antioxidant status/capacity status, and oxidative stress. As antioxidants can act additively or synergistically, and absorbed and used in the human body in different ways, so the judgment based on total antioxidant activity provides more credible data rather than the quantification of one antioxidant individually. These include indices that focus the total scavenging abilities of serum sample, following, for example, addition of a radical forming compound. So, we used the most common tests for evaluation including FRAP and DPPH assays.

Our findings demonstrated that the subjects with a higher FQS (greater consuming of dietary fiber, MUFA, vitamin B6, vitamin C, magnesium, Fe, vegetables, fruits, legumes and nuts, and whole grains) were more likely to have lower blood levels of WBC, RDW, NLR, PLR. A long with us, Fung et al. (2005) reported that Alternate Healthy Eating Index (AHEI), and aMED which more emphasize on DPs rich in fruit, vegetables, nuts, whole grains, and sea foods are significantly correlated with lower indices of inflammation including serum CRP and IL‐6 in middle aged women. In consistent, a DP high in sugar and fat has been connected with low‐grade inflammation identified by high concentrations of CRP (Liu et al., 2002). There is increasing evidence that a healthier diet or health‐protecting foods with anti‐inflammatory contents, such as high intakes of grains and fiber, vegetables, fruit (Lopez‐Garcia et al., 2004), MUFA (Fraker et al., 2000), Fe, magnesium (Oppenheimer, 2001), and vitamin C (Garcia‐Diaz et al., 2014), are inversely associated with serum inflammatory mediators. It has been shown that intake of nuts causes to decreased levels of inflammation and cholesterol; therefore cardioprotective effects throughout the body (Ros, 2015). Although, no associations was observed between diet quality indices with inflammatory biomarkers, such as hs‐CRP and IL‐6, in youth diabetic patients (Liese et al., 2018).

Food quality score is a validated tool to capture the overall diet quality according to food intake which provides valuable estimation about the DPs of individuals or populations and health status. This approach focuses on total diet rather than using a single food‐intake determination approach. We controlled for important potential confounder variables that could affect the relationships. Moreover, energy‐adjusted intakes of FQS items were used, which can decrease the risk of misclassification of study subjects. But, present study has several limitations. In epidemiological studies, evaluation of diet depends on self‐reported information which is susceptible to inevitable recall bias. Also, even though urinary FRAP, DPPH and MDA levels were adjusted by urinary creatinine, these amounts were measured by spot urine specimens. Finally, the cross‐sectional nature of this study does not make it conceivable to disclose a causality effect.

## 5 CONCLUSION

Overall, our data suggest that consuming a good quality diet characteristically rich in fruits, vegetables, nuts, and whole grain and low in discretionary foods is associated with lower values of inflammatory markers and higher anti‐oxidant capacity biomarkers in young women. Future studies will attempt on underlying mechanisms. Moreover, future dietary modification studies may provide a public health knowledge on whether diet modification interventions may be beneficial in combating pathologies through targeting oxidative stress and inflammation.

## AUTHOR CONTRIBUTIONS

AB was involved in conceptualization, data curation, formal analysis, funding acquisition, investigation, methodology, project administration, supervision, and writing—original draft preparation. FN was involved in investigation and methodology. ZK was involved in resources and software. MM was involved in validation and visualization. GF was involved in conceptualization (supporting), data curation (supporting), and writing—review and editing. All of the authors have read and confirmed the final manuscript.

## FUNDING INFORMATION

This study was supported by grants from Birjand University of Medical Sciences, Birjand, Iran.

## CONFLICT OF INTEREST

The authors declare that they have no conflict of interest.

## ETHICAL APPROVAL

Ethical approval was obtained from the Birjand University of Medical Sciences and informed written consent was completed by all participants (code: IR.BUMS.REC.1398.402).

## INFORMED CONSENT

Informed consent was obtained from all individual participants included in the study.

## CONSENT FOR PUBLICATION

Not applicable.

## References

[phy215590-bib-0001] Abbaszadeh, A. , Saharkhiz, M. , Khorasanchi, Z. , Karbasi, S. , Askari, M. , Hoseini, Z. S. , Ayadilord, M. , Mahmoudzadeh, S. , Rezapour, H. , & Enayati, H. (2020). Impact of a Nordic diet on psychological function in young students. Nutrition and Health, 27, 97–104. 33076738 10.1177/0260106020964981

[phy215590-bib-0002] Ahmadnezhad, M. , Asadi, Z. , Miri, H. H. , Ferns, G. A. , Ghayour‐Mobarhan, M. , & Ebrahimi‐Mamaghani, M. (2017). Validation of a short semi‐quantitative food frequency questionnaire for adults: A pilot study. Journal of Nutritional Sciences and Dietetics, 3, 49–55.

[phy215590-bib-0003] Aleksandrova, K. , Koelman, L. , & Rodrigues, C. E. (2021). Dietary patterns and biomarkers of oxidative stress and inflammation: A systematic review of observational and intervention studies. Redox Biology, 42, 101869. 33541846 10.1016/j.redox.2021.101869 PMC8113044

[phy215590-bib-0004] Askari, M. , Abbaszadeh, A. , Saharkhiz, M. , Karbasi, S. , Talebpour, A. , Fashami, A. A. A. , Rezapour, H. , Hoseini, Z. S. , Mahmoudzadeh, S. , & Ayadilord, M. (2020). A study of the association between cognitive abilities and dietary intake in young women. Nutrition and Health, 26, 263–270. 32646288 10.1177/0260106020940116

[phy215590-bib-0005] Bandyopadhyay, U. , Das, D. , & Banerjee, R. K. (1999). Reactive oxygen species: Oxidative damage and pathogenesis. Current Science, 77, 658–666.

[phy215590-bib-0006] Bao, Y. , Yang, M. , Jin, C. , Hou, S. , Shi, B. , Shi, J. , & Lin, N. (2018). Preoperative hematologic inflammatory markers as prognostic factors in patients with glioma. World Neurosurgery, 119, e710–e716. 30092479 10.1016/j.wneu.2018.07.252

[phy215590-bib-0007] Bärebring, L. , Winkvist, A. , Gjertsson, I. , & Lindqvist, H. M. (2018). Poor dietary quality is associated with increased inflammation in Swedish patients with rheumatoid arthritis. Nutrients, 10, 1535. 30340316 10.3390/nu10101535 PMC6213361

[phy215590-bib-0008] Benzie, I. F. , & Strain, J. J. (1996). The ferric reducing ability of plasma (FRAP) as a measure of “antioxidant power”: The FRAP assay. Analytical Biochemistry, 239, 70–76. 8660627 10.1006/abio.1996.0292

[phy215590-bib-0009] Cao, G. , Russell, R. M. , Lischner, N. , & Prior, R. L. (1998). Serum antioxidant capacity is increased by consumption of strawberries, spinach, red wine or vitamin C in elderly women. The Journal of Nutrition, 128, 2383–2390. 9868185 10.1093/jn/128.12.2383

[phy215590-bib-0010] Chen, X. , Touyz, R. M. , Park, J. B. , & Schiffrin, E. L. (2001). Antioxidant effects of vitamins C and E are associated with altered activation of vascular Nadph oxidase and superoxide dismutase in stroke‐prone Shr. Hypertension, 38, 606–611. 11566940 10.1161/hy09t1.094005

[phy215590-bib-0011] Darooghegi Mofrad, M. , Namazi, N. , Larijani, B. , Bellissimo, N. , & Azadbakht, L. (2019). The association of food quality score and cardiovascular diseases risk factors among women: A cross‐sectional study. Journal of Cardiovascular and Thoracic Research, 11, 237–243. 31579465 10.15171/jcvtr.2019.39 PMC6759612

[phy215590-bib-0012] Fraker, P. J. , King, L. E. , Laakko, T. , & Vollmer, T. L. (2000). The dynamic link between the integrity of the immune system and zinc status. The Journal of Nutrition, 130, 1399S–1406S. 10801951 10.1093/jn/130.5.1399S

[phy215590-bib-0013] Fu, H. , Qin, B. , Hu, Z. , Ma, N. , Yang, M. , Wei, T. , Tang, Q. , Huang, Y. , Huang, F. , & Liang, Y. (2015). Neutrophil‐and platelet‐to‐lymphocyte ratios are correlated with disease activity in rheumatoid arthritis. Clinical Laboratory, 61, 269–273. 25974992 10.7754/clin.lab.2014.140927

[phy215590-bib-0014] Fung, T. T. , Mccullough, M. L. , Newby, P. , Manson, J. E. , Meigs, J. B. , Rifai, N. , Willett, W. C. , & Hu, F. B. (2005). Diet‐quality scores and plasma concentrations of markers of inflammation and endothelial dysfunction. The American Journal of Clinical Nutrition, 82, 163–173. 16002815 10.1093/ajcn.82.1.163

[phy215590-bib-0015] Fung, T. T. , Pan, A. , Hou, T. , Mozaffarian, D. , Rexrode, K. M. , Willett, W. C. , & Hu, F. B. (2016). Food quality score and the risk of coronary artery disease: A prospective analysis in 3 cohorts. The American Journal of Clinical Nutrition, 104, 65–72. 27281310 10.3945/ajcn.116.130393 PMC4919530

[phy215590-bib-0016] Garcia‐Diaz, D. F. , Lopez‐Legarrea, P. , Quintero, P. , & Martinez, J. A. (2014). Vitamin C in the treatment and/or prevention of obesity. Journal of Nutritional Science and Vitaminology, 60, 367–379. 25866299 10.3177/jnsv.60.367

[phy215590-bib-0017] Hassimotto, N. M. A. , Pinto, M. R. D. S. , & Lajolo, F. M. (2008). Antioxidant status in humans after consumption of blackberry (*Rubus fruticosus* L.) juices with and without defatted milk. Journal of Agricultural and Food Chemistry, 56, 11727–11733. 19053224 10.1021/jf8026149

[phy215590-bib-0018] Ho, S. , Chen, Y. , Woo, J. , Leung, S. , Lam, T. , & Janus, E. (2001). Association between simple anthropometric indices and cardiovascular risk factors. International Journal of Obesity, 25, 1689–1697. 11753592 10.1038/sj.ijo.0801784

[phy215590-bib-0019] Hosseini, F. , Shab‐Bidar, S. , Ghanbari, M. , Majdi, M. , Sheikhhossein, F. , & Imani, H. (2021). Food quality score and risk of breast cancer among Iranian women: Findings from a case control study. Nutrition and Cancer, 74, 1660–1669. 34323136 10.1080/01635581.2021.1957136

[phy215590-bib-0020] Hu, Z. , Chen, H. , Huang, L. , Chen, S. , Huang, Z. , Qin, S. , Zhong, J. , Qin, X. , & Li, S. (2019). Correlation between hematological parameters and ancylostomiasis: A retrospective study. Journal of Clinical Laboratory Analysis, 33, e22705. 30390342 10.1002/jcla.22705 PMC6818622

[phy215590-bib-0021] Hu, Z.‐D. , Chen, Y. , Zhang, L. , Sun, Y. , Huang, Y.‐L. , Wang, Q.‐Q. , Xu, Y.‐L. , Chen, S.‐X. , Qin, Q. , & Deng, A.‐M. (2013). Red blood cell distribution width is a potential index to assess the disease activity of systemic lupus erythematosus. Clinica Chimica Acta, 425, 202–205. 10.1016/j.cca.2013.08.007 23954839

[phy215590-bib-0022] Jacob, K. , Periago, M. J. , Böhm, V. , & Berruezo, G. R. (2008). Influence of lycopene and vitamin C from tomato juice on biomarkers of oxidative stress and inflammation. British Journal of Nutrition, 99, 137–146. 17640421 10.1017/S0007114507791894

[phy215590-bib-0023] Janaszewska, A. , & Bartosz, G. (2002). Assay of total antioxidant capacity: Comparison of four methods as applied to human blood plasma. Scandinavian Journal of Clinical and Laboratory Investigation, 62, 231–236. 12088342 10.1080/003655102317475498

[phy215590-bib-0024] Kamińska, J. , Koper, O. M. , Siedlecka‐Czykier, E. , Matowicka‐Karna, J. , Bychowski, J. , & Kemona, H. (2018). The utility of inflammation and platelet biomarkers in patients with acute coronary syndromes. Saudi Journal of Biological Sciences, 25, 1263–1271. 30505168 10.1016/j.sjbs.2016.10.015 PMC6252018

[phy215590-bib-0025] Kei, S. (1978). Serum lipid peroxide in cerebrovascular disorders determined by a new colorimetric method. Clinica Chimica Acta, 90, 37–43. 10.1016/0009-8981(78)90081-5 719890

[phy215590-bib-0026] Kim, J. , Yang, Y. , Yang, Y. , Oh, S. , Hong, Y. , Lee, E. , & Kwon, O. (2011). Diet quality scores and oxidative stress in Korean adults. European Journal of Clinical Nutrition, 65, 1271–1278. 21712839 10.1038/ejcn.2011.120

[phy215590-bib-0027] Kruk, J. , Aboul‐Enein, H. Y. , Kładna, A. , & Bowser, J. E. (2019). Oxidative stress in biological systems and its relation with pathophysiological functions: The effect of physical activity on cellular redox homeostasis. Free Radical Research, 53, 497–521. 31039624 10.1080/10715762.2019.1612059

[phy215590-bib-0028] Lavigne‐Robichaud, M. , Moubarac, J.‐C. , Lantagne‐Lopez, S. , Johnson‐Down, L. , Batal, M. , Sidi, E. A. L. , & Lucas, M. (2018). Diet quality indices in relation to metabolic syndrome in an Indigenous Cree (Eeyouch) population in northern Québec, Canada. Public Health Nutrition, 21, 172–180. 28683844 10.1017/S136898001700115X PMC10260753

[phy215590-bib-0029] Lee, J. H. , Ralston, R. A. , & Truby, H. (2011). Influence of food cost on diet quality and risk factors for chronic disease: A systematic review. Nutrition and Dietetics, 68, 248–261.

[phy215590-bib-0030] Liese, A. D. , Ma, X. , Ma, X. , Mittleman, M. A. , The, N. S. , Standiford, D. A. , Lawrence, J. M. , Pihoker, C. , Marcovina, S. M. , & Mayer‐Davis, E. J. (2018). Dietary quality and markers of inflammation: No association in youth with type 1 diabetes. Journal of Diabetes and its Complications, 32, 179–184. 29198994 10.1016/j.jdiacomp.2017.10.015 PMC5773064

[phy215590-bib-0031] Liu, S. , Manson, J. E. , Buring, J. E. , Stampfer, M. J. , Willett, W. C. , & Ridker, P. M. (2002). Relation between a diet with a high glycemic load and plasma concentrations of high‐sensitivity C‐reactive protein in middle‐aged women. The American Journal of Clinical Nutrition, 75, 492–498. 11864854 10.1093/ajcn/75.3.492

[phy215590-bib-0032] Lopes, H. F. , Martin, K. L. , Nashar, K. , Morrow, J. D. , Goodfriend, T. L. , & Egan, B. M. (2003). Dash diet lowers blood pressure and lipid‐induced oxidative stress in obesity. Hypertension, 41, 422–430. 12623938 10.1161/01.HYP.0000053450.19998.11

[phy215590-bib-0033] Lopez‐Garcia, E. , Schulze, M. B. , Fung, T. T. , Meigs, J. B. , Rifai, N. , Manson, J. E. , & Hu, F. B. (2004). Major dietary patterns are related to plasma concentrations of markers of inflammation and endothelial dysfunction. The American Journal of Clinical Nutrition, 80, 1029–1035. 15447916 10.1093/ajcn/80.4.1029

[phy215590-bib-0034] Miller Iii, E. R. , Erlinger, T. P. , Sacks, F. M. , Svetkey, L. P. , Charleston, J. , Lin, P.‐H. , & Appel, L. J. (2005). A dietary pattern that lowers oxidative stress increases antibodies to oxidized Ldl: Results from a randomized controlled feeding study. Atherosclerosis, 183, 175–182. 16216596 10.1016/j.atherosclerosis.2005.04.001

[phy215590-bib-0035] Oppenheimer, S. J. (2001). Iron and its relation to immunity and infectious disease. The Journal of Nutrition, 131, 616S–635S. 11160594 10.1093/jn/131.2.616S

[phy215590-bib-0036] Paz, M. F. C. J. , Gomes Júnior, A. L. , De Alencar, M. V. O. B. , Tabrez, S. , Islam, M. T. , Jabir, N. R. , Oves, M. , Alam, M. Z. , Asghar, M. N. , & Ali, E. S. (2019). Effect of diets, familial history, and alternative therapies on genomic instability of breast cancer patients. Applied Biochemistry and Biotechnology, 188, 282–296. 30430345 10.1007/s12010-018-2918-9

[phy215590-bib-0037] Ros, E. (2015). Nuts and CVD. British Journal of Nutrition, 113, S111–S120. 26148914 10.1017/S0007114514003924

[phy215590-bib-0038] Stewart, R. , Askew, E. , Mcdonald, C. , Metos, J. , Jackson, W. , Balon, T. , & Prior, R. (2002). Antioxidant status of young children: Response to an antioxidant supplement. Journal of the American Dietetic Association, 102, 1652–1657. 12449290 10.1016/s0002-8223(02)90352-4

[phy215590-bib-0039] Thorpe, M. G. , Kestin, M. , Riddell, L. J. , Keast, R. S. , & Mcnaughton, S. A. (2014). Diet quality in young adults and its association with food‐related behaviours. Public Health Nutrition, 17, 1767–1775. 23866858 10.1017/S1368980013001924 PMC10282490

[phy215590-bib-0040] Tsang, C. , Smail, N. F. , Almoosawi, S. , Davidson, I. , & Al‐Dujaili, E. A. (2012). Intake of polyphenol‐rich pomegranate pure juice influences urinary glucocorticoids, blood pressure and homeostasis model assessment of insulin resistance in human volunteers. Journal of Nutritional Science, 1, e9. 25191556 10.1017/jns.2012.10 PMC4153032

[phy215590-bib-0041] Ushula, T. W. , Lahmann, P. H. , Mamun, A. , Wang, W. Y. , Williams, G. M. , & Najman, J. M. (2021). Lifestyle correlates of dietary patterns among young adults: Evidence from an Australian birth cohort. Public Health Nutrition, 25, 2167–2178. 10.1017/S1368980021003864 PMC9991692 34486516

[phy215590-bib-0042] Waijers, P. M. , Feskens, E. J. , & Ocké, M. C. (2007). A critical review of predefined diet quality scores. British Journal of Nutrition, 97, 219–231. 17298689 10.1017/S0007114507250421

[phy215590-bib-0043] Wang, L. , Jia, J. , Lin, L. , Guo, J. , Ye, X. , Zheng, X. , & Chen, Y. (2017). Predictive value of hematological markers of systemic inflammation for managing cervical cancer. Oncotarget, 8, 44824–44832. 28148894 10.18632/oncotarget.14827 PMC5546522

[phy215590-bib-0044] Yang, M. , Ma, N. , Fu, H. , Wei, T. , Tang, Q. , Qin, B. , Yang, Z. , & Zhong, R. (2015). Hematocrit level could reflect inflammatory response and disease activity in patients with systemic lupus erythematosus. Clinical Laboratory, 61, 801–807. 26299080 10.7754/clin.lab.2015.141246

[phy215590-bib-0045] Yang, W. , Wang, X. , Zhang, W. , Ying, H. , Xu, Y. , Zhang, J. , Min, Q. , & Chen, J. (2017). Neutrophil‐lymphocyte ratio and platelet‐lymphocyte ratio are 2 new inflammatory markers associated with pulmonary involvement and disease activity in patients with dermatomyositis. Clinica Chimica Acta, 465, 11–16. 10.1016/j.cca.2016.12.007 27965019

